# Enhanced nerve function recovery in radial nerve palsy patients with humerus shaft fracture: a randomized study of low-frequency pulse electrical stimulation combined with exercise therapy

**DOI:** 10.3389/fneur.2024.1370316

**Published:** 2024-07-01

**Authors:** Shaoyan Shi, Xuehai Ou, Xiaolong Du

**Affiliations:** Honghui Hospital, Xi'an Jiaotong University, Xi'an, China

**Keywords:** humerus fracture, radial nerve injury, low-frequency pulse electrical stimulation, exercise therapy, electromyography

## Abstract

**Objective:**

To evaluate the effect of low-frequency pulse electrical stimulation plus exercise therapy on nerve function recovery in patients with radial nerve palsy after humerus shaft fracture.

**Methods:**

A total of 110 patients with humerus shaft fracture and radial nerve injury admitted to our hospital from January 2017 to December 2021 were recruited. They were randomized to receive either conventional exercise therapy (control group) or conventional exercise therapy plus low-frequency pulse electrical stimulation (study group) according to the random number table method, with 55 cases in each. Clinical efficacy, muscle strength recovery, nerve conduction velocity (MCV), amplitude, wrist joint, and elbow joint activities of patients were analyzed and compared.

**Results:**

Patients with low frequency stimulation (LFS) showed significantly higher treatment effectiveness (89.09%) than those with exercise therapy only (69.09%). The incorporation of LFS with exercise therapy provided more enhancement in the muscle strength of wrist extensor and total finger extensor in patients when compared with a mere exercise intervention, suggesting better muscle function recovery of patients produced by LFS. Moreover, a significant increase in MCV and its amplitude was observed in all included patients, among which those receiving LFS showed a greater escalation of MCV and its amplitude. Following a treatment duration of 6 months, more patients in the LFS cohort were reported to achieve a wrist extension and elbow extension with an angle over 45° than the controls. There was no notable variance in adverse responses noted between the two patient groups.

**Conclusion:**

In patients afflicted with humerus shaft fracture and radial nerve injury, the amalgamation of exercise therapy with low-frequency pulse electrical stimulation can significantly improve clinical efficacy, promote nerve function, and muscle strength recovery, and features a high safety profile.

**Relevance to clinical practice:**

The combination of exercise therapy and low-frequency pulsed electrical stimulation can notably improve the promotion of neurologic function and muscle strength recovery in patients with humerus shaft fractures and radial nerve injuries with a high degree of safety.

**Clinical trial registration:**https://www.researchregistry.com, identifier researchregistry9461.

## 1 Introduction

Peripheral nerve injuries accompanied by fractures are predominantly reported in the upper limbs, constituting approximately 95% of all cases of fractures with peripheral nerve damage. Among these, radial nerve injuries associated with humeral shaft fractures represent the most common scenario (1). Humeral shaft fractures account for about 3% of all orthopedic injuries (2). Radial nerve palsy (RNP) is a common complication of humeral shaft fractures and is classified into primary RNP and secondary RNP (3). Primary RNP refers to nerve injuries potentially induced by localized compression at the fracture site, transverse disruption of fracture fragments, or local swelling at the moment of the fracture (4). Secondary RNP, also known as iatrogenic RNP, develops as a result of medical treatment or intervention, accounting for 10%–20% of all humeral shaft fracture-related RNPs (5). The occurrence of radial nerve injuries is closely associated with its anatomical location. The close proximity to the humeral shaft in the middle and lower third renders the radial nerve susceptible to injury in cases of humeral shaft fractures. Excessive callus formation, radial head dislocation, and inadvertent surgical trauma can also lead to radial nerve compression (6). Recent retrospective studies have reported an incidence of radial nerve injuries associated with humeral shaft fractures ranging from 11% to 18% (7).

Damage to the radial nerve can result in deficiencies in both motor and sensory capacities, chiefly marked by the inability to perform wrist, thumb, and finger extensions, coupled with numbness on the dorsal side of the hand. Recovery from radial nerve injuries tends to be protracted, fueling an ongoing discourse about their clinical treatment strategies (8). The primary objectives in managing radial nerve palsy involve addressing muscle weakness, preventing muscular wasting, enhancing the overall quality of life, and minimizing risks of disability (9). Low-frequency pulsed electrical stimulation, as a clinically commonly used rehabilitation treatment, is often combined with other drug therapies to promote the recovery of elderly patients with intertrochanteric hip fractures (10), and has shown good clinical efficacy in promoting the recovery of limb function in children with brachial plexus nerve injuries (11). Traditional approaches to humeral shaft fractures with concomitant radial nerve injuries typically involve conservative treatment, but the treatment outcomes are incongruent across studies. Low-frequency pulse electrical stimulation therapy refers to a treatment approach that involves applying electrical currents with a low pulse frequency, typically ranging from 1 to 10 Hz or lower, to the body for therapeutic purposes (12). In recent years, with the continuous advancement of medical devices, Devices utilizing low-frequency pulse electrical stimulation therapy have been progressively incorporated into the clinical treatment of diverse medical conditions, exhibiting encouraging outcomes across a spectrum of medical disciplines (13). In the current study, a combination of physical exercise and low-frequency pulse electrical stimulation was applied and yielded positive outcomes in cases of humeral shaft fractures with radial nerve injuries.

## 2 Materials and methods

### 2.1 Study subjects and design

Our Institutional Review Board approved this single-center, randomized, double-blinded, parallel controlled trial (project approval number researchregistry9461), registered at ClinicalTrials.gov.^1^ The study was carried out in the Honghui Hospital, Xi’an Jiaotong University, from January 2017 to December 2021. A total of 110 patients with humerus shaft fracture and radial nerve injury admitted to our hospital from January 2017 to December 2021 were recruited and randomized to receive either conventional exercise therapy (control group) or conventional exercise therapy plus low-frequency pulse electrical stimulation (study group) according to the random number table method, with 55 cases in each group. Before patients are officially enrolled, neither the experimenter nor the participants can know the specific grouping situation. Before enrollment, subjects were informed about the study protocol and provided informed consent. All procedures in this study adhered to the ethical principles of the Helsinki Declaration regarding clinical research and were approved by our hospital’s ethics committee (no. 2021092106).

### 2.2 Inclusion and exclusion criteria

Inclusion criteria: (1) Confirmed humeral shaft fracture by X-ray or other examinations; (2) Received fracture treatment at our hospital; (3) Radial nerve injury confirmed by electromyography: (1) Complete injury: There is spontaneous electrical activity, but there is no spontaneous electrical activity in MUP (Motor Unit Potential). CMAP (Compound Muscle Action Potential), SNAP (Sensory Nerve Action Potential), and MNCV (Motor Nerve Conduction Velocity) all disappear. (2) Severe injury: There is spontaneous electrical activity, but no spontaneous electrical activity in MUP. The amplitude of CMAP decreases, SNAP decreases or disappears, and MNCV slows down or disappears. (3) Incomplete injury: There is prolongation of spontaneous electrical activity or insertion potential, reduction of MUP, decrease in CMAP and SNAP, while MNCV remains normal or decelerates (14, 15). (4) Age between 18 and 80 years; (5) Patients with initial fractures.

Exclusion criteria: (1) Neural compression is not relieved, and nerve rupture is not repaired; (2) Other peripheral nerve injuries, such as ulnar nerve injury or median nerve injury; (3) Uncontrolled diseases that affect postoperative recovery, such as hypertension, diabetes, or hyperlipidemia; (4) Coagulation disorders, immune dysfunction, or similar conditions; (5) Communication barriers or mental disorders; (6) Poor compliance, unable to cooperate with the rehabilitation treatment plan or unable to accept postoperative follow-up.

### 2.3 Intervention measures

Upon admission, all patients received intramuscular injections of nerve growth factor at a dose of 15,000 U daily, along with oral administration of 10 mg of vitamin B1, thrice daily, and 500 μg of methylcobalamin, thrice daily, for 30 days. During the study period, patients in both groups did not take other neurotrophic drugs. Patients with open fractures underwent surgical treatment, including exploration of the elbow blood vessels and nerves. If there was a combination of blood vessel and nerve rupture, the stump was treated, and anastomosis was performed after stable fixation with Kirschner wires at the elbow. The control group received exercise therapy, while the study group received low-frequency pulse electrical stimulation therapy combined with exercise therapy. Based on the patient’s tolerance, exercise therapy should be conducted 1 to 2 times per day. The same physicians administered the treatment to the individuals and assessed their clinical scores.

Exercise therapy encompassed both active and passive exercises. Passive exercises involved gently performing slow joint movements to achieve a normal range of motion. For the affected limb, gentle pressing and tapping of acupoints such as Hegu, Quchi, and Waiguan were conducted, accompanied by a light brushing of the active muscle surfaces in the direction of joint movement. Active exercises comprised wrist extension and finger extension exercises, progressively transitioning to muscle contraction exercises and resistance training. Exercise therapy was started the day after fracture surgery. The intervention lasted for 2 months, gradually transitioning from passive to active exercises.

Low frequency pulsed electrical stimulation was used for low frequency pulsed electrical stimulation. The process involved the use of two electrodes (4 cm × 8 cm) placed in a water-soaked sponge bag, secured onto the extensor digitorum brevis, extensor hallucis longus, and radial wrist extensor muscles on the affected side. The positive electrode was positioned in proximity to the site of radial nerve injury, while the negative electrode was placed distally.

The stimulation of finger and wrist extension was achieved by adjusting the current intensity on the device unit, ranging from 2 to 19.5 mA, with a frequency of 2 to 10 Hz, using the Keypoint four-channel electromyography evoked potential instrument (Dantec Dynamics, Denmark). A biphasic current with symmetrical waveform was delivered continuously for 15 s, with a rise time of 3 s and a rest period of 30 s. The pulse duration was set at 250 μs. The intensity was individually adjusted by each subject to their maximum tolerable limit and applied four times per session on the affected limb, with a total intervention period of 2 months.

### 2.4 Outcome measures

#### 2.4.1 Clinical efficacy

After 6 months of treatment, the nerve function improvement rates of the two groups were compared. Evaluation was conducted based on the functional criteria for repaired radial nerve injuries, which were categorized as significantly effective, effective, less effective, or ineffective. The evaluation criteria for radial nerve function refer to the “Trial Standards for Functional Evaluation of Upper Limbs of the Chinese Medical Association Hand Surgery Society” (16). Specifically, significantly effective was defined as a score of 13 to 16, effective as a score of 9 to 12, less effective as a score of 5 to 8, and ineffective as a score of ≤4. The clinical effective rate was calculated as follows: Clinical effective rate = (significantly effective + effective)/Total number of cases × 100.00%.

#### 2.4.2 Electromyography results

Prior to treatment and after 6 months of treatment, electromyographic (EMG) indicators of extensor digitorum and wrist extensor muscle strength recovery were assessed and compared using an electromyography apparatus for both groups of patients. The measurement of muscle strength referred to the muscle strength observation indicators established by Wang Rao et al. (17). Electromyography indicators of muscle strength recovery of the extensor digitorum communis and extensor carpi radialis longus muscles in the two groups of patients were detected and compared using electromyography before and after treatment.

Additionally, needle electrodes were inserted into the muscle belly of the wrist extensor and extensor digitorum muscles to serve as 9033G0701 electromyograph (Dantec Dynamics, Denmark). Stimulation potentials were applied at sites including the biceps brachii and triceps brachii intramuscular heads near the axilla, as well as at the Erb’s point near the suprascapular nerve, long thoracic nerve, and distal to the dorsal scapular nerve. This procedure allowed for the measurement of nerve conduction velocity (MCV) and amplitude of the EMG signals.

#### 2.4.3 Wrist and elbow extension angles

After 6 months of treatment, wrist and elbow extension angles were measured. The range of motion (ROM) of normal active movements of the wrist and elbow joints was measured. The normal ROM for wrist flexion and extension is from 60° dorsiflexion to 60° palmar flexion. The functional position for lateral deviation is 0°~10°, and the normal ROM for wrist lateral deviation ranges from 20° radial deviation to 30° ulnar deviation. The normal ROM for the elbow joint is as follows: flexion from 0° to 150°, extension from 0° to 10° hyperextension, pronation from 0° to 90°, and supination from 0° to 90°. An angle greater than 60° was classified as excellent, 45°–60° as good, and less than 45° as poor (16).

#### 2.4.4 Complications

All patients were followed up continuously for 2 months, with weekly visits to record and compare the occurrence rates of infections, joint pain, and delayed fracture healing during the treatment period.

### 2.5 Statistical analysis

Participants did not know which group they were assigned to. The staff member responsible for sample selection was unaware of the specific implementation plan for each group. The staff member responsible for data collection was unaware of the group assignments and the specific implementation plan for each group. The researcher was aware of the three specific implementation plans but did not know the group assignments. Data were processed and analyzed using SPSS 22.0 software, and figures were generated using GraphPad Prism 9.0. Measurement data were expressed as mean ± standard deviation and compared between groups using the independent samples t-test. Count data were expressed as rates and compared between groups using the chi-square test. A significance level of α = 0.05 was used to determine statistical significance.

## 3 Results

### 3.1 Baseline patient profiles

All subjects were included in the observation, and there were no dropouts during the study period. All patients completed follow-up visits after surgery. The comparison of baseline characteristics between the two patient groups is presented in Table 1. In the control group, there were 36 males and 19 females, with an average age of 48.26 ± 7.15 years and an average duration of illness of 4.25 ± 1.05 weeks. Among them, 22 cases had left-sided injuries and 33 cases had right-sided injuries. The distribution of fractures included 38 cases of mid-upper humeral fractures, 17 cases of humeral condyle fractures, 9 cases of open fractures, and 46 cases of closed fractures. In the observation group, there were 32 males and 23 females, with an average age of 46.95 ± 9.21 years and an average duration of illness of 4.11 ± 1.17 weeks. Among them, 27 cases had left-sided injuries and 28 cases had right-sided injuries. The distribution of fractures included 34 cases of mid-upper humeral fractures, 21 cases of humeral condyle fractures, 11 cases of open fractures, and 44 cases of closed fractures. The two groups were well-balanced in terms of baseline patient profiles (*p* > 0.05), indicating comparability.

**Table 1:** Patient characteristics.

	Control group	Study group	χ2/t	*p*-value
*n*	55	55		
Age ( $\bar{x}$ ±s, years old)	48.26 ± 7.15	46.95 ± 9.21	0.833	0.407
Gender (*n*, %)			0.616	0.432
Male	36	32		
Female	19	23		
Disease course ( $\bar{x}$ ±s, years weeks)	4.25 ± 1.05	4.11 ± 1.17	0.660	0.510
Fracture side (*n*, %)			0.920	0.338
Left	22	27		
Right	33	28		
Fracture location (*n*, %)			0.643	0.423
Middle and lower part	38	34		
Supracondylar	17	21		
Type of injury			0.244	0.621
Open fractures	9	11		
Closed fractures	46	44		

### 3.2 Clinical efficacy

In the control group, there were 11 cases with significantly effective outcomes, 27 cases with effective outcomes, 10 cases with less effective outcomes, and 7 cases with ineffective outcomes, yielding a clinical effective rate of 69.09% (38/55). In the study group, 23 cases with significantly effective outcomes, 26 cases with effective outcomes, 4 cases with less effective outcomes, and 2 cases with ineffective outcomes, yielding a clinical effective rate of 89.09% (49/55). Patients with low frequency stimulation (LFS) showed significantly higher treatment effectiveness (89.09%) than those with exercise therapy only (69.09; Table 2; *p* < 0.05).

**Table 2:** Comparison of clinical efficacy.

	*n*	Significantly effective	Effective	Less effective	Ineffective	Effective rate
Control group	55	11	27	10	7	38 (69.09%)
Study group	55	23	26	4	2	49 (89.09%)
χ2						6.652
*P*						0.01

### 3.3 Comparison of wrist extensor and finger extensor muscle strength

**Figure 1:**
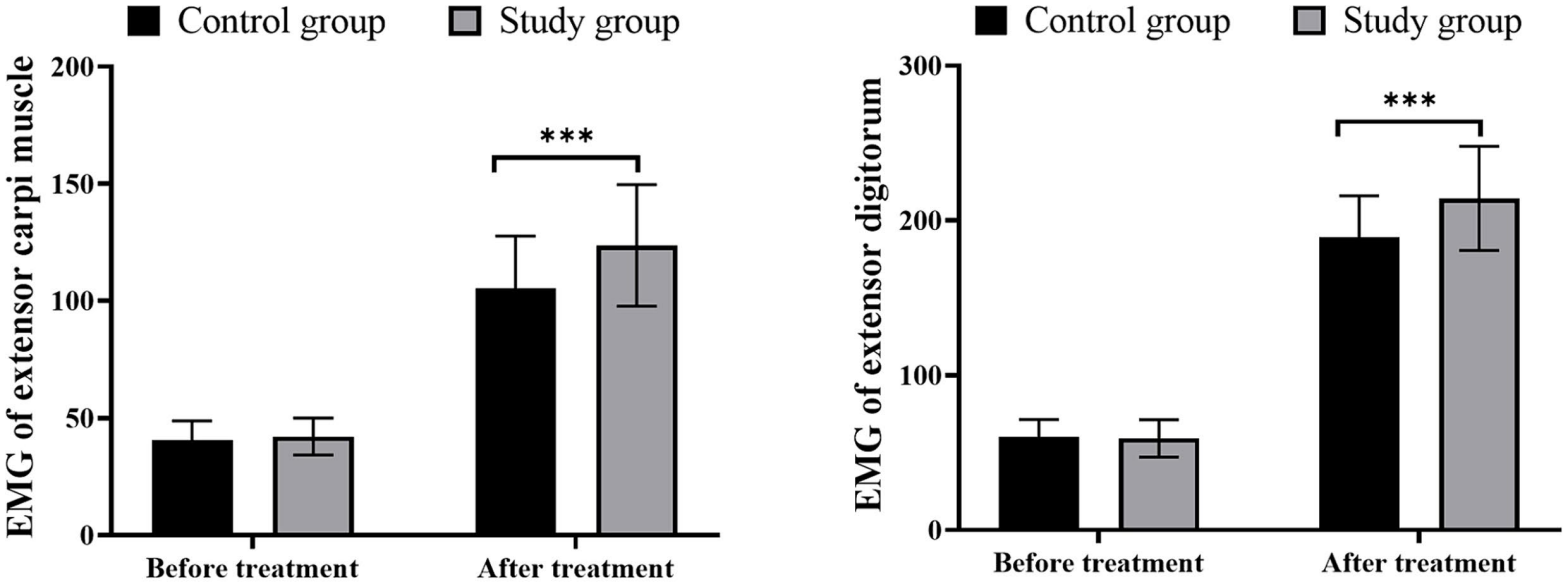
Comparison of wrist extensor and finger extensor muscle strength, ^***^p < 0.001.

Before treatment, there were no significant differences in wrist extensor and finger extensor muscle strength between the two groups (*p* > 0.05). The incorporation of LFS with exercise therapy provided more enhancement in the muscle strength of wrist extensor and total finger extensor in patients when compared with mere exercise intervention, suggesting better muscle function recovery of patients produced by LFS (Figure 1; *p* < 0.05).

### 3.4 Comparison of nerve conduction velocity and amplitude

**Figure 2:**
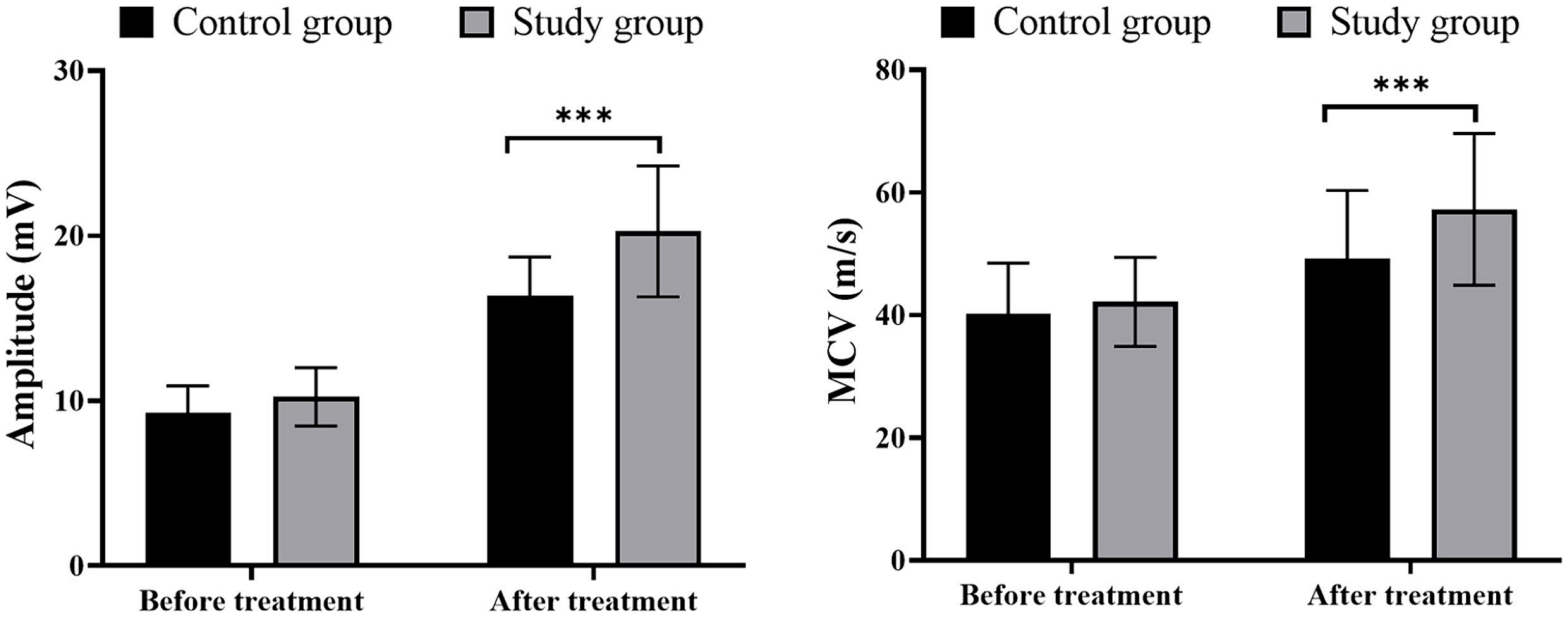
Comparison of nerve conduction velocity and amplitude, ^***^p < 0.001.

Prior to treatment, no significant differences were observed in MCV and amplitude between the two groups (*p* > 0.05). Moreover, a significant increase in MCV and its amplitude was observed in all included patients, among which those receiving LFS showed a greater escalation of MCV and its amplitude (Figure 2; *p* < 0.05).

### 3.5 Comparison of wrist and elbow extension angles

In terms of wrist extension angles, in the control group, there were 16 cases rated as “excellent,” 23 cases as “good,” and 16 cases as “poor.” In the study group, there were 30 cases rated as “excellent,” 19 cases as “good,” and 6 cases as “poor.” As for elbow extension angles, In the control group, there were 18 cases rated as “excellent,” 20 cases as “good,” and 17 cases as “poor.” In the study group, there were 26 cases rated as “excellent,” 24 cases as “good,” and 5 cases as “poor.” Following a treatment duration of 6 months, more patients in the LFS cohort were reported to achieve a wrist extension and elbow extension with an angle over 45° than the controls (Tables 3, 4; *p* < 0.05).

**Table 3:** Comparison of wrist extension angles.

	*n*	Excellent	Good	Poor	Effective rate
Control group	55	16	23	16	39 (70.91%)
Study group	55	30	19	6	49 (89.09%)
χ2					5.682
*P*					0.017

**Table 4:** Comparison of elbow extension angles.

	*n*	Excellent	Good	Poor	Effective rate
Control group	55	18	20	17	38 (69.09%)
Study group	55	26	24	5	50 (90.91%)
χ2					8.182
*P*					0.004

### 3.6 Comparison of complications during follow-up

In the control group, there were 2 cases of incisional infection, 1 case of joint pain, 1 case of delayed fracture healing, and 1 case of other complications, resulting in a total complication rate of 9.09% (5/55). In the study group, there were 3 cases of incisional infection, 2 cases of joint pain, and 2 cases of other complications, yielding a total complication rate of 12.73% (7/55). No significant difference in adverse reactions was observed between the two groups of patients (*p* > 0.05; Table 5).

**Table 5:** Comparison of complications during follow-up.

	*n*	Incisional infection	Joint pain	Delayed fracture healing	Other complications	Incidence of complications
Control group	55	2	1	1	1	5 (9.09%)
Study group	55	3	2	0	2	7 (12.73%)
*χ* ^2^						0.374
*P*						0.541

## 4 Discussion

The radial nerve, the largest nerve in the upper limb, originates from the posterior cord of the brachial plexus and gives off nerve branches. Its fibers arise from the C5-8 spinal nerve roots. The radial nerve is situated within the spiral groove of the humerus shaft and is frequently jeopardized following humeral shaft fractures (18). Radial nerve injuries are often transient and can be managed with conservative treatments such as rest, functional exercises, nonsteroidal anti-inflammatory drugs, and functional bracing. Radial nerve injury represents a prevalent category within peripheral nerve injuries. Despite the regenerative capabilities possessed by damaged axons, their functional recovery is often suboptimal. Without neuronal contact, injured nerves progressively lose their regenerative capacity in a time-sensitive and length-dependent manner. Schwann cells, which lose nerve innervation, eventually atrophy and cannot support axon regeneration (19). Thus, it is vital to provide appropriate treatment strategies that facilitate the reconnection of nerves in the affected area and enhance the speed of nerve regeneration (20). The prognosis of humeral shaft fractures combined with radial nerve injury varies with the degree of injury, the location of the damage, and most patients’ symptoms resolve within a few weeks. However, severe cases may persist for months, years, or even lifetime (21).

In the present study, low-frequency pulse electrical stimulation therapy was incorporated with exercise therapy for radial nerve palsy following humeral shaft fractures, resulting in significant benefits in muscle strength recovery, elbow and wrist extension angles, as well as nerve conduction velocity and amplitude. Exercise therapy is the fundamental treatment approach for humeral shaft fractures complicated with radial nerve injury, primarily aimed at restoring the functional movement of relevant muscles. Early mobilization of affected joints and muscles is necessary to prevent tendon adhesions and joint stiffness. Nevertheless, the impact of exercise therapy alone is constrained (22). Low-frequency pulse electrical stimulation therapy is an emerging clinical treatment modality that can effectively regulate neurophysiological activities, inhibit nerve degeneration, and promote nerve regeneration (23). Previous research has shown that low-frequency pulse electrical stimulation therapy has positive effects on patients with median nerve injury caused by carpal tunnel release surgery, accelerating axon regeneration and muscle nerve innervation without compromising the functional recovery of carpal tunnel syndrome patients (12). Research has demonstrated that low-frequency pulse electrical stimulation therapy can promote M2 macrophage expression, activate early steps in muscle regeneration, and accelerate collagen synthesis, thereby enhancing nerve repair speed (24). Furthermore, low-frequency stimulation therapy induces Ca2+ influx, leading to upregulation of brain-derived neurotrophic factor (BDNF) and tyrosine kinase receptor B expression in nerve cells (25). Overexpression of BDNF inhibits phosphodiesterase activity, causing sustained increases in cAMP levels, activating cyclic AMP response element-binding protein (CREB), upregulating regeneration-associated genes (RAGs) such as tubulin-alpha-1 and growth-associated protein-43 (GAP-43) expression, and suppressing Rho to accelerate cytoskeletal formation, thereby enhancing nerve regeneration (26, 27). In addition to nerve cells, Schwann cells are also affected by electrical stimulation. In a tibial nerve transection animal model, brief electrical stimulation during surgery was used to improve axon regeneration of transplanted nerves (28). Combined treatment of low-frequency pulse electrical stimulation therapy with exercise therapy can promote nerve cell regeneration, increase nerve blood flow, provide a favorable nutritional environment for nerve regeneration, and promote nerve function recovery. This contributes to the restoration of normal joint activity, leading to significantly higher clinical effectiveness, muscle strength recovery, wrist extension angle, and elbow extension angle efficacy in the study group compared to the control group.

The results of this study suggest that the excellent rate of neurological recovery in the combined group was 89.09%, which was higher than that in the control group (69.09%; *p* < 0.05). The reason lies in the fact that electrical stimulation improves blood circulation in the affected limb, thereby accelerating the exchange of nutrients needed for nerve repair. Low-frequency pulsed electrical stimulation is beneficial for the proliferation and differentiation of bone cells, thereby accelerating bone healing. For the local swelling and bleeding that occur after a fracture, goal-directed rehabilitation therapy can promote soft tissue recovery and relieve pain at the fracture site. In addition, it can promote fracture healing, facilitate callus connection, and enhance bone density to some extent. Furthermore, Gordon et al. (29) demonstrated that impaired nerve regeneration is associated with transient expression of growth-related genes, leading to a decline in the regenerative capacity of neurons and Schwann cells over time. However, low-frequency electrical stimulation has been shown to expedite the growth of motor and sensory axons at the site of injury. This effect has been observed in both animal models and patients even when there is a delay in surgical repair of the injured nerve. Electrical stimulation enhances nerve regeneration and promotes target reinnervation, as it elevates the levels of neuronal cyclic adenosine monophosphate (cAMP), thereby increasing the expression of neurotrophic factors and other growth-related genes, including cytoskeletal proteins. Future research should focus on the mechanism of action of low-frequency pulsed electrical stimulation on bone cells, as well as its effects on ions such as calcium, magnesium, and phosphorus in the human body. Neurological rehabilitation is a relatively long process, and apart from the efforts of medical workers, the level of patient cooperation is also one of the important factors that determine the final rehabilitation outcome. Low-frequency pulsed electrical therapy can alleviate patient pain, promote neurological recovery, and accelerate bone healing, showing significant clinical effects.

This study has the following limitations. Firstly, in terms of the selection of research subjects, objective assessment of nerve injury location and severity is scarce. Secondly, only clinical outcomes and EMG results were included, lacking investigations on underlying mechanisms. Thirdly, the follow-up period was relatively short, and long-term effectiveness remains unclear.

## 5 Conclusion

In patients afflicted with humerus shaft fracture and radial nerve injury, the amalgamation of exercise therapy with low-frequency pulse electrical stimulation can significantly improve clinical efficacy, promote nerve function and muscle strength recovery, and features a high safety profile. For patients with humeral shaft fractures combined with radial nerve injury, the combination of exercise therapy and low-frequency pulsed electrical stimulation can significantly improve clinical efficacy, promote the recovery of nerve function and muscle strength, thereby helping to improve patient compliance with rehabilitation treatment, reduce the risk of complications, and have practical significance in reducing patients’ secondary hospitalization plans and saving medical costs.

## Data availability statement

The original contributions presented in the study are included in the article/supplementary material, further inquiries can be directed to the corresponding author.

## Ethics statement

The studies involving humans were approved by the Ethics Committee of the Honghui Hospital, Xi’an Jiaotong University. The number of the Ethics Committee’s acceptance is: (2021092106). The studies were conducted in accordance with the local legislation and institutional requirements. The participants provided their written informed consent to participate in this study.

## Author contributions

SS: Conceptualization, Formal analysis, Funding acquisition, Investigation, Resources, Software, Supervision, Validation, Visualization, Writing – original draft, Writing – review & editing. XO: Conceptualization, Data curation, Formal analysis, Funding acquisition, Investigation, Methodology, Resources, Software, Supervision, Validation, Writing – original draft, Writing – review & editing. XD: Conceptualization, Data curation, Formal analysis, Investigation, Methodology, Project administration, Resources, Software, Supervision, Validation, Visualization, Writing – original draft, Writing – review & editing.

## Footnotes

^1^https://www.researchregistry.com

## References

[1] Ciaramitaro P Mondelli M Logullo F Grimaldi S Battiston B Sard A. Traumatic peripheral nerve injuries: epidemiological findings, neuropathic pain and quality of life in 158 patients. J Peripher Nerv Syst. (2010) 15:120–7. doi: 10.1111/j.1529-8027.2010.00260.x 20626775

[2] Steffner RJ Lee MA. Emerging concepts in upper extremity trauma: humeral shaft fractures. Orthop Clin North Am. (2013) 44:21–33. doi: 10.1016/j.ocl.2012.08.005 23174323

[3] Van Bergen SH Mahabier KC Van Lieshout EMM Van der Torre T Notenboom CAW Jawahier PA. Humeral shaft fracture: systematic review of non-operative and operative treatment. Arch Orthop Trauma Surg. (2023) 143:5035–54. doi: 10.1007/s00402-023-04836-8 37093269 PMC10374687

[4] Daly M Langhammer C. Radial nerve injury in humeral shaft fracture. Orthop Clin North Am. (2022) 53:145–54. doi: 10.1016/j.ocl.2022.01.001 35365259

[5] DeFranco MJ Lawton JN. Radial nerve injuries associated with humeral fractures. J Hand Surg Am. (2006) 31:655–63. doi: 10.1016/j.jhsa.2006.02.013 16632062

[6] Hendrickx LAM Hilgersom NFJ Alkaduhimi H Doornberg JN van den Bekerom MPJ. Radial nerve palsy associated with closed humeral shaft fractures: a systematic review of 1758 patients. Arch Orthop Trauma Surg. (2021) 141:561–8. doi: 10.1007/s00402-020-03446-y 32285189 PMC7966639

[7] Shao YC Harwood P Grotz MR Limb D Giannoudis PV. Radial nerve palsy associated with fractures of the shaft of the humerus: a systematic review. J Bone Joint Surg Br. (2005) 87-B:1647–52. doi: 10.1302/0301-620X.87B12.16132 16326879

[8] Niver GE Ilyas AM. Management of radial nerve palsy following fractures of the humerus. Orthop Clin North Am. (2013) 44:419–24. doi: 10.1016/j.ocl.2013.03.012 23827843

[9] Pet MA Lipira AB Ko JH. Nerve transfers for the restoration of wrist, finger, and thumb extension after high radial nerve injury. Hand Clin. (2016) 32:191–207. doi: 10.1016/j.hcl.2015.12.007 27094891

[10] Tienan Q Daorui G Jinxian P Shuhao C. Treatment of intertrochanteric fracture of femur in the elderly with teriparatide combined with low frequency pulsed electromagnetic fields. Chin J Joint Surg. (2019) 13:760–4. doi: 10.3877/cma.j.issn.1674-134X.2019.06.019

[11] Junmei L. The effect of early application of low-frequency pulsed electrical stimulation combined with cerebroside and ganglioside on the rehabilitation efficacy of children with brachial plexus injury. Nurs Res. (2019) 33:720–1. doi: 10.12102/j.issn.1009-6493.2019.04.047

[12] Ehnert S Schröter S Aspera-Werz RH Eisler W Falldorf K Ronniger M. Translational insights into extremely low frequency pulsed electromagnetic fields (ELF-PEMFs) for bone regeneration after trauma and orthopedic surgery. J Clin Med. (2019) 8:2028. doi: 10.3390/jcm8122028 31756999 PMC6947624

[13] Iijima H Takahashi M Tashiro Y Aoyama T. Comparison of the effects of kilohertz- and low-frequency electric stimulations: a systematic review with meta-analysis. PLoS One. (2018) 13:e0195236. doi: 10.1371/journal.pone.0195236 29689079 PMC5915276

[14] Zhang Kaili Xu Jianguang F Clinically practical neuromuscular Electrodiagnostic techniques. Shanghai: Fudan University Press, (2004). 101–118.

[15] Jingxia Dang. Electromyography diagnosis and clinical application. Beijing: People's Medical Publishing House, (2005). 215–222.

[16] Dade P Yudong G De S Kuishui S. Trial standards for functional evaluation of upper limbs of the Chinese Medical Association hand surgery society. Chin J Hand Surg. (2000) 3:4–9. doi: 10.3760/cma.j.issn.1005-054X.2000.03.003

[17] Rao W Zhuo Y Lihua W Yun Y Zhenzhen Z Yin Z. Study on the influence of electromyography characteristics combined with exercise therapy on electromyography and motor nerve conduction velocity in patients with radial nerve injury due to upper Limb fracture. China Med Equip. (2020) 17:79–82. doi: 10.3969/J.ISSN.1672-8270.2020.07.020

[18] Liang S Liu YZ Hu XQ Zhao X Lao J. Restoration of intrinsic hand function by superficial radial nerve: an anatomical study. BMC Musculoskelet Disord. (2023) 24:628. doi: 10.1186/s12891-023-06758-3 37532990 PMC10394765

[19] Elzinga K Tyreman N Ladak A Savaryn B Olson J Gordon T. Brief electrical stimulation improves nerve regeneration after delayed repair in Sprague Dawley rats. Exp Neurol. (2015) 269:142–53. doi: 10.1016/j.expneurol.2015.03.022 25842267

[20] Ni L Yao Z Zhao Y Zhang T Wang J Li S. Electrical stimulation therapy for peripheral nerve injury. Front Neurol. (2023) 14:1081458. doi: 10.3389/fneur.2023.1081458 36908597 PMC9998520

[21] Orapiriyakul W Apivatthakakul V Theppariyapol B Apivatthakakul T. Humerus shaft fractures, approaches and management. J Clin Orthop Trauma. (2023) 43:102230. doi: 10.1016/j.jcot.2023.102230 37588079 PMC10425411

[22] Hegeman EM Polmear M Scanaliato JP Nesti L Dunn JC. Incidence and Management of Radial Nerve Palsies in humeral shaft fractures: a systematic review. Cureus. (2020) 12:e11490. doi: 10.7759/cureus.11490 33335819 PMC7736027

[23] Chang G Ilyas AM. Radial nerve palsy after humeral shaft fractures: the case for early exploration and a new classification to guide treatment and prognosis. Hand Clin. (2018) 34:105–12. doi: 10.1016/j.hcl.2017.09.011 29169591

[24] Reidy PT Edvalson LT McKenzie AI Petrocelli JJ Mahmassani ZS Drummond MJ. Neuromuscular electrical stimulation and protein during bed rest increases CD11b(+) skeletal muscle macrophages but does not correspond to muscle size or insulin sensitivity. Appl Physiol Nutr Metab. (2020) 45:1261–9. doi: 10.1139/apnm-2020-0064 32470312 PMC9236569

[25] Geremia NM Gordon T Brushart TM Al-Majed AA Verge VM. Electrical stimulation promotes sensory neuron regeneration and growth-associated gene expression. Exp Neurol. (2007) 205:347–59. doi: 10.1016/j.expneurol.2007.01.040 17428474

[26] Cobianchi S Casals-Diaz L Jaramillo J Navarro X. Differential effects of activity dependent treatments on axonal regeneration and neuropathic pain after peripheral nerve injury. Exp Neurol. (2013) 240:157–67. doi: 10.1016/j.expneurol.2012.11.023 23201096

[27] Han PJ Shukla S Subramanian PS Hoffman PN. Cyclic AMP elevates tubulin expression without increasing intrinsic axon growth capacity. Exp Neurol. (2004) 189:293–302. doi: 10.1016/j.expneurol.2004.03.010 15380480

[28] Keane GC Pan D Roh J Larson EL Schellhardt L Hunter DA. The effects of intraoperative electrical stimulation on regeneration and recovery after nerve Isograft repair in a rat model. Hand. (2022) 17:540–8. doi: 10.1177/1558944720939200 32666827 PMC9112755

[29] Gordon T. Electrical stimulation to enhance axon regeneration after peripheral nerve injuries in animal models and humans. Neurotherapeutics. (2016) 13:295–310. doi: 10.1007/s13311-015-0415-1 26754579 PMC4824030

